# Population‐level inferences from environmental DNA—Current status and future perspectives

**DOI:** 10.1111/eva.12882

**Published:** 2019-11-18

**Authors:** Eva Egelyng Sigsgaard, Mads Reinholdt Jensen, Inger Eleanor Winkelmann, Peter Rask Møller, Michael Møller Hansen, Philip Francis Thomsen

**Affiliations:** ^1^ Department of Bioscience Aarhus University Aarhus C Denmark; ^2^ Natural History Museum of Denmark University of Copenhagen Copenhagen Ø Denmark

**Keywords:** aquatic, environmental DNA, high‐throughput sequencing, noninvasive sampling, population genomics

## Abstract

Environmental DNA (eDNA) extracted from water samples has recently shown potential as a valuable source of population genetic information for aquatic macroorganisms. This approach offers several potential advantages compared with conventional tissue‐based methods, including the fact that eDNA sampling is noninvasive and generally more cost‐efficient. Currently, eDNA approaches have been limited to single‐marker studies of mitochondrial DNA (mtDNA), and the relationship between eDNA haplotype composition and true haplotype composition still needs to be thoroughly verified. This will require testing of bioinformatic and statistical software to correct for erroneous sequences, as well as biases and random variation in relative sequence abundances. However, eDNA‐based population genetic methods have far‐reaching potential for both basic and applied research. In this paper, we present a brief overview of the achievements of eDNA‐based population genetics to date, and outline the prospects for future developments in the field, including the estimation of nuclear DNA (nuDNA) variation and epigenetic information. We discuss the challenges associated with eDNA samples as opposed to those of individual tissue samples and assess whether eDNA might offer additional types of information unobtainable with tissue samples. Lastly, we provide recommendations for determining whether an eDNA approach would be a useful and suitable choice in different research settings. We limit our discussion largely to contemporary aquatic systems, but the advantages, challenges, and perspectives can to a large degree be generalized to eDNA studies with a different spatial and temporal focus.

## 1 INTRODUCTION

Population genetic and genomic studies of aquatic macroorganisms can be logistically challenging, resource‐demanding, and potentially harmful to the study organisms (Hansen, 1988; Pirhonen & Schreck, 2003) as well as their habitats (e.g., bottom trawling, see Fosså, Mortensen, and Furevik (2002) and Jørstad (2004)). In particular, the physical collection of tissue samples from individuals can be difficult for elusive species, such as those living in the deep sea (Winkelmann et al., 2013) or hidden inside coral reefs (Brandl, Goatley, Bellwood, & Tornabene, 2018). Sampling and international transport of tissue samples also involve extensive permit requirements, especially when working with protected species. Analysis of environmental DNA (eDNA) isolated from water samples has already been established as a noninvasive and cost‐efficient tool for species detection (Evans, Shirey, Wieringa, Mahon, & Lamberti, 2017; Ficetola, Miaud, Pompanon, & Taberlet, 2008; Sigsgaard, Carl, Møller, & Thomsen, 2015; Thomsen, Kielgast, Iversen, Wiuf, et al., 2012), but has more recently also shown great promise for obtaining population genetic information (Adams et al., 2019; Baker, Steel, Nieukirk, & Klinck, 2018; Gorički et al., 2017; Parsons, Everett, Dahlheim, & Park, 2018; Sigsgaard et al., 2016; Stat et al., 2017; Stepien, Snyder, & Elz, 2019; Uchii, Doi, & Minamoto, 2016) (Table 1). Collection of eDNA from water samples is nondestructive, it is resource‐ and time‐efficient, and it offers a larger “catch‐window” than traditional sampling approaches, by detecting individuals that are not necessarily present at the exact time and place of sampling, but are present in the overall study area (Baker et al., 2018).

**Table 1:** An overview of eDNA studies that have obtained population‐level information

Reference	Environment	Target taxon/taxa	Variant detection approach	Mitochondrial target gene(s)/region(s)	Length of marker(s) (bp)
Uchii et al. (2016)	Freshwater	*Cyprinus carpio* (common carp)	qPCR	D‐loop	240
Sigsgaard et al. (2016)	Marine	*Rhincodon typus* (whale shark)	Species‐level metabarcoding	D‐loop	412–493
Gorički et al. (2017)	Freshwater	*Proteus anguinus* (olm)	qPCR	D‐loop, cytochrome b, and 16S rRNA	106–157
Stat et al. (2017)	Marine	Fishes	Multispecies metabarcoding	16S rRNA	178–228
Parsons et al. (2018)	Marine	*Phocoena phocoena* (harbour porpoise)	Species‐level metabarcoding	Cytochrome b	160
Baker et al. (2018)	Marine	*Orcinus orca* (killer whale)	ddPCR	D‐loop	139–246
Marshall and Stepien (2019)	Freshwater	*Dreissena polymorpha* and *D. rostriformis* (Eurasian zebra and quagga mussels)	Multispecies metabarcoding	Cytochrome oxidase I	169–175
Stepien et al. (2019)	Freshwater	*Hypophthalmichthys molitrix *(silver carp)	Multispecies metabarcoding	Cytochrome b	135
Turon et al. (2019)	Marine	Eukaryotes	Multispecies metabarcoding	Cytochrome oxidase I	313

Note

The type of aquatic environment, taxa and genetic region(s) targeted, as well as the technique applied for detection of genetic variation, and the size of the targeted markers are given.

Sigsgaard et al. (2016) demonstrated that eDNA from seawater samples can provide information on intraspecific genetic diversity through DNA metabarcoding (Taberlet, Bonin, Zinger, & Coissac, 2018; Taberlet, Coissac, Hajibabaei, & Rieseberg, 2012) of a short marker in the D‐loop (control region) of the mitochondrial genome (Table 1). Based on this marker, inferences on haplotype diversity, population structure, and female effective population size were made and were found to conform well with results obtained with conventional tissue‐based analyses (Sigsgaard et al., 2016). This single‐marker approach thus presents an attractive way of obtaining basic population‐level insights. However, by leveraging molecular laboratory techniques from other fields, for example, ancient DNA research (Der Sarkissian et al., 2015), eDNA from water samples could potentially provide even deeper and broader insights into aquatic macroorganismal populations, rivaling those obtainable from tissue samples. Most importantly, for eDNA research to attain its full potential within molecular ecology, the field needs to progress from mitochondrial eDNA to leveraging the much higher‐resolution information contained within nuclear DNA. In this article, we begin by summarizing which biological aspects of wild populations are currently being studied using population genetic and genomic methods based on traditional tissue samples, then briefly review the current state of eDNA‐based population genetic research, and lastly, we discuss whether and how additional population‐level information might be obtained from eDNA samples in the future. We limit our scope largely to the study of contemporary, macroorganismal eDNA from water samples (i.e., we do not discuss bulk or fecal samples of aquatic organisms), which is a type of eDNA sampling that has become widely used within the last decade. Nevertheless, the potential applications are directly applicable to other eDNA or bulk‐tissue DNA sample types, including ancient eDNA, and to other taxonomic groups.

## 2 WHICH POPULATION CHARACTERISTICS CAN CURRENTLY BE ESTIMATED WITH GENETIC TECHNIQUES?

A wide range of insights into the biology of a population can be obtained by studying genetic diversity. This includes characteristics pertaining to the composition of the population at the time of sampling, such as sex ratio (e.g., Dallas et al., 2003), kinship between individuals (e.g., Patel, Thompson, Santure, Constantine, & Millar, 2017), and census population size as estimated by genetic capture–mark–recapture (e.g., Citta et al., 2018). Genetic data can also provide insights into a population's demographic and evolutionary history. This can be achieved through estimation of the effective population size, N_e_ (Waples, 1989), demographic history reconstruction (Luikart, Sherwin, Steele, & Allendorf, 1998), or analyses of intra‐ and interspecific hybridization, introgression (e.g., Takahashi et al., 2016), and secondary contact (Tine et al., 2014). It can also be done by testing for connectivity (Lowe & Allendorf, 2010; Waples & Gaggiotti, 2006) or differing patterns of selection in separate populations (Williams & Oleksiak, 2011). Differential selection between populations is often related to spatial habitat delimitation, determined by either biotic (such as the presence of certain predators (Richardson & Urban, 2013)) or abiotic (such as salinity, see Fietz et al. (2018) and Nielsen, Nielsen, Meldrup, and Hansen (2004), or temperature, see Bradbury et al. (2010)) factors that restrict dispersal (Selkoe et al., 2016). Over the last decade, population genetics has entered the genomic era, and marine populations are now increasingly being studied within the framework of landscape (or seascape) genomics (Selkoe et al., 2016; Xuereb, Kimber, Curtis, Bernatchez, & Fortin, 2018). In this framework, researchers test for correlations between genome‐wide variation and a range of oceanographic (currents, eddies, etc.) and environmental (temperature, salinity, etc.) parameters, in order to understand the mechanisms behind population differentiation and to identify selection regimes possibly affecting individual loci (Nielsen, Hemmer‐Hansen, Larsen, & Bekkevold, 2009; Nielsen, 2005). In a more applied example of population genomic research, outlier scans can be used to identify loci with the most power for discriminating between populations (Gagnaire et al., 2015; Nielsen et al., 2012). These loci can subsequently be used for population assignment tests to determine the origin of specific individuals, for instance in catches of commercially exploited fish species (Knutsen et al., 2018; Nielsen et al., 2012).

Many studies have used markers in the mitochondrial genome to study population structure (e.g., Baker et al., 1993; Encalada et al., 1996; Taguchi, King, Wetklo, Withler, & Yokawa, 2015) and effective population size (e.g., Castro et al., 2007; Hrbek et al., 2005). However, the mitochondrial genome constitutes only a single evolutionarily independent locus for such analyses, because mitochondrial DNA (mtDNA) very rarely recombines, at least in higher animals (although see Ciborowski et al., 2007; Ujvari, Dowton, & Madsen, 2007). Mitochondria are usually exclusively maternally inherited (although see, e.g., Luo et al., 2018), and the resulting smaller effective population size of the mitochondrial genome in a given population, compared with that of the nuclear genome, can lead to contrasting patterns of genetic differentiation in mtDNA and nuclear genotypes (Birky, Maruyama, & Fuerst, 1983). Such incongruence can also occur due to sex‐specific differences in dispersal (e.g., Karl, Castro, Lopez, Charvet, & Burgess, 2011; Tillett et al., 2012). See also Prugnolle and de Meeus (2002) for a review on inferring sex‐biased dispersal using population genetic tools. Gene conversion (Lapierre, Blin, Lambert, Achaz, & Rocha, 2016) and the interacting effects of demography and selection on genetic variation (Williamson et al., 2005) may also render analyses of demographic history and selection problematic when applied to mtDNA in isolation. Lastly, mtDNA may not always live up to the key statistical assumption that it evolves under neutral selection (Ballard & Kreitman, 1995; Consuegra, John, Verspoor, & Leaniz, 2015), rendering results based on certain types of evolutionary models unreliable. To obtain data that are more robust, and to include information from both sexes, it is now common practice to include nuclear DNA (nuDNA) in population genetic studies (for a comprehensive review of the key differences between mtDNA and nuDNA, see Ballard & Whitlock, 2004). Techniques for investigating nuDNA have historically progressed from the use of allozymes (Harris, 1966; Kojima, Gillespie, & Tobari, 1970), to microsatellite‐based approaches (Jarne & Lagoda, 1996; Li, Korol, Fahima, Beiles, & Nevo, 2002; Zane, Bargelloni, & Patarnello, 2002), and with the advent of high‐throughput sequencing (HTS), either reduced representation libraries (RRL) (Altshuler et al., 2000; Elshire et al., 2011; Vignal, Milan, SanCristobal, & Eggen, 2002) or whole‐genome sequencing (WGS) (The *Arabidopsis* Genome Initiative, 2000; The *C. elegans* Sequencing Consortium, 1998), depending on the research question and available budget. Naturally, WGS is the gold standard, as it provides the most comprehensive datasets, allowing for a deeper understanding of population history. However, factors such as large and/or complex genomes, the need for a certain minimum sample size (of sequenced individuals) for robust statistical analyses, and poor starting DNA quality are often prohibitive (Wandeler, Hoeck, & Keller, 2007; Weisrock et al., 2018) to this approach. This often leads researchers to employ RRL methods, where short genetic regions across the nuclear genome are sequenced, yielding a large number of (more or less) independent sites for comparisons across individuals and populations, while retaining the option of including a large number of individuals (Baird et al., 2008; Davey et al., 2011).

## 3 POPULATION GENETIC STUDIES BASED ON ENVIRONMENTAL DNA

Over the last three decades, traditional tissue sampling for population genetics has increasingly been supplemented by noninvasive genetic sampling via the collection of alternative genetic materials, such as feces (e.g., Bellemain, Swenson, Tallmon, Brunberg, & Taberlet, 2005; Höss, Kohn, Pääbo, Knauer, & Schröder, 1992; Prigioni et al., 2006) or hair (e.g., Mowat & Strobeck, 2000; Taberlet, Mattock, Dubois‐Paganon, & Bouvet, 1993; Valiere et al., 2003). In 2003, it was shown for the first time that DNA from past communities of macrofauna and flora could be detected in sediment samples (Willerslev et al., 2003), and since then, a variety of environmental samples such as ice (Willerslev et al., 2007), air (Kraaijeveld et al., 2015), soil (Yoccoz et al., 2012; Zinger et al., 2018), and especially water (Ficetola et al., 2008; Jerde, Mahon, Chadderton, & Lodge, 2011; Stat et al., 2017; Thomsen, Kielgast, Iversen, Møller, et al., 2012; Thomsen, Kielgast, Iversen, Wiuf, et al., 2012) samples have been used to detect a wide range of macroorganisms from both past and present ecosystems (Taberlet et al., 2018; Thomsen & Willerslev, 2015). Due to the fact that historical or ancient eDNA, as well as eDNA from some modern sample types, is almost invariably degraded and fragmented, the eDNA approach has mainly relied on DNA barcodes designed to be as short as possible (<100–150 bp in length for highly degraded DNA and seldom longer than ~250 bp), while simultaneously retaining the highest possible resolution for taxonomic identification (Taberlet et al., 2018). Thus, the first study (to the best of our knowledge) to apply eDNA from water samples to study intraspecific genetic diversity used a marker that was just long enough to cover one single nucleotide polymorphism (SNP) and thus discriminate between the native and non‐native populations of a freshwater fish species (Uchii et al., 2016) (Table 1). A study by Gorički et al. (2017) similarly used markers of ~100 and ~150 bp to distinguish between two color morphs of the cave‐dwelling amphibian *Proteus anguinus* (Table 1). However, recently shed eDNA from living organisms may also be present in the form of complete cells or long DNA fragments (Deiner et al., 2017). Thus, Sigsgaard et al. (2016) demonstrated that eDNA from water samples contained sufficiently long and abundant mtDNA fragments that metabarcoding markers covering multiple polymorphisms can be applied, allowing for more detailed population genetic analyses.

The highly variable D‐loop of the mitochondrial genome can provide key population‐level information, and using *Rhincodon typus* Smith, 1828 (the whale shark), as a model organism, Sigsgaard et al. (2016) provided evidence that this genetic information can be obtained directly from seawater samples. Mitochondrial D‐loop haplotypes from the eDNA samples matched known haplotypes from whale shark tissue samples, and crucially, the relative abundance of eDNA haplotypes corresponded well with tissue‐based estimates of haplotype frequencies in the studied aggregation. Parsons et al. (2018) applied a similar approach to a population of *Phocoena phocoena* (Linnaeus, 1758) (harbour porpoise) (Table 1) and also found that the D‐loop haplotypes obtained from eDNA samples matched known haplotypes of harbour porpoise and reflected previous estimates of relative haplotype frequencies. Additionally, the spatial distribution of haplotypes in the eDNA samples indicated phylogeographic structure within the studied population (Parsons et al., 2018). Baker et al. (2018) applied droplet digital PCR, also targeting the D‐loop region, to distinguish between different ecotypes of *Orcinus orca* (Linnaeus, 1758) (killer whale) (Table 1), and found that eDNA results were consistent with hydrophone recordings and visual observations. Most recently, Stepien et al. (2019) applied a marker in the mtDNA cytochrome *b* (Cyt*b*) gene to distinguish between specific haplotypes of the invasive *Hypophthalmichthys molitrix* (Valenciennes, 1844) (silver carp), while Marshall and Stepien (2019) used a region of the mtDNA cytochrome c oxidase I (COI) gene to distinguish between different haplotypes within the invasive mussel species *Dreissena polymorpha* (Pallas (1771)) (European zebra mussel) and *D. rostriformis* (Deshayes, 1838) (quagga mussel) (Table 1). Stat et al. (2017) obtained information on intraspecific diversity within fish species of the genus *Lethrinus* through metabarcoding of the mitochondrial large ribosomal subunit (16S) gene of fishes (Table 1).

## 4 FUTURE POTENTIAL FOR POPULATION GENETIC ANALYSES OF MITOCHONDRIAL ENVIRONMENTAL DNA

While mtDNA provides only one independent marker for population genetic studies, a major advantage for eDNA studies is that the mtDNA genome exists in several copies in each cell (Bogenhagen & Clayton, 1974) and that mtDNA appears to degrade at a slower rate than nuDNA (Allentoft et al., 2012; Schwarz et al., 2009). Therefore, the chances of the target eDNA being sufficiently abundant and sufficiently intact (long) for successful detection are expected to be greater than for nuDNA. Another important advantage of mtDNA is its prior application in population genetics and DNA barcoding, which means that there is an extended reference database compared with many nuclear genes. The mtDNA markers used by Sigsgaard et al. (2016) and Parsons et al. (2018) ranged from ~ 400 to almost 500 bp in length, and Deiner et al. (2017) recently showed that complete mitochondrial genomes can be amplified directly from eDNA using long‐range PCR. These results support the hypothesis that not all macroorganismal eDNA is highly degraded, and suggest that intact macroorganismal cells, or at least complete organelles (e.g., mitochondria), likely contribute to the accessible eDNA pool. Advancements in high‐throughput sequencing technologies, such as 600 bp paired‐end sequencing by synthesis (SBS) on the MiSeq™ (Illumina Inc.) system and third‐generation sequencing technologies (reviewed by van Dijk, Jaszczyszyn, Naquin, & Thermes, 2018), including single‐molecule real‐time sequencing (SMRT) (Levene et al., 2003) and nanopore sequencing (Cherf et al., 2012; Manrao et al., 2012), hold great promise for future studies targeting long eDNA fragments. These kinds of technologies would allow for the generation of high‐resolution mitochondrial haplotype data, and potentially analyses of demographic history and selection using, for example, Bayesian Skyline Plots (Heled & Drummond, 2008) and Tajima (1983). While the evidence published to date supports a good correlation between the relative abundances of eDNA sequences and the relative abundances of the species or haplotypes they originate from (Parsons et al., 2018; Sigsgaard et al., 2016; Thomsen, Møller, et al., 2016), further pilot experiments are needed to systematically test whether (or when) this holds true across different environments and target organisms. In this context, an advantage of sequencing longer mtDNA reads would be that the measured haplotype richness could potentially provide a reasonable estimate of the number of individuals present, which would likely be a more robust quantification than relying on eDNA copy number or read counts (Evans et al., 2016; Shelton et al., 2016; Thomsen, Møller, et al., 2016). Last, but not least, the portability of the nanopore sequencing device MinION™ (Oxford Nanopore Technologies) allows for high‐throughput long‐read sequencing of eDNA samples in the field, which offers great convenience for eDNA studies in remote places and for faster and simpler workflows.

## 5 FUTURE POTENTIAL FOR POPULATION GENETIC ANALYSES OF NUCLEAR ENVIRONMENTAL DNA

As mentioned above, mtDNA only allows for partial insights into a population's history and evolution, and we hypothesize that if intact macroorganismal cells are indeed present in environmental samples, then these should contain sufficient amounts of nuDNA for genome‐wide population genetic analyses. Copy numbers of nuDNA markers in an eDNA sample will, with the possible exception of multi‐copy regions (such as rRNA genes), be significantly lower than those of mtDNA, and further optimization of current field and laboratory protocols may therefore be required, including the collection of larger water sample volumes and development of more efficient eDNA extraction protocols. It will also be essential to tailor the sampling regime as specifically as possible to the area(s) where the species of interest is known (or expected) to be present. Such “targeted sampling” can be based on prior knowledge of the species' distribution, ecology, and behavior (Sigsgaard et al., 2015), on data from satellite tags or echo sounders, or on direct observations (Baker et al., 2018; Parsons et al., 2018; Sigsgaard et al., 2016). This may well be challenging for certain organisms, but if sufficient amounts of nuDNA can be collected with such approaches, it would allow for much more detailed and accurate population genetic analyses than those done with mtDNA alone.

### 5.1 Genome‐wide approaches for determining, for example, population structure, demographic history, and selection

It should be noted that for one popular category of RRL sequencing, namely those methods that rely on restriction enzyme digestion (such as genotyping by sequencing (GBS, Elshire et al., 2011), restriction site‐associated DNA sequencing (RADseq, Baird et al., 2008), and double digest RADseq (ddRADseq) (Peterson, Weber, Kay, Fisher, & Hoekstra, 2012)), it is a fundamental requirement that the starting material contains high molecular weight endogenous DNA. This is rarely the case for eDNA samples, and additionally, the indiscriminate frequent‐cutter nature of the restriction enzymes used in such methods is likely to cut any and all DNA molecules present in the sample, be they of target or nontarget origin, possibly leading to sequencing of such large amounts of undesirable fragments that the genetic signal from the target organism(s) is drowned out. Fortunately, a different and more targeted RRL approach can be applied, where most of the nontarget DNA present in the sample is removed before sequencing. This method is known as target enrichment via DNA hybridization capture, often referred to simply as target capture. The technique can involve targeting specific genes or genomic regions of interest as in, for example, exome sequencing (Teer & Mullikin, 2010), or targeting loci previously identified with other RRL methods (e.g., Ali et al., 2016). Relatively small amounts of starting DNA material are required for this approach (Gnirke et al., 2009; Hodges et al., 2007), which has made it advantageous in studies where endogenous DNA content is low and fragment size is small, such as in ancient samples (Enk et al., 2014). Recently, target capture of mtDNA has been applied to both ancient eDNA from sediment (Slon et al., 2017) and modern eDNA from water samples (Mariac et al., 2018; Wilcox et al., 2018) for species detection. Although a reference genome is required to design capture probes for population genomic analyses, this genome may be sourced from a related species (even a distant one), since a perfect match between the probes and the target DNA is not a strict necessity (Enk et al., 2014). After complementary eDNA sequences have hybridized to the capture probes, the remaining nontarget DNA molecules are flushed away (or kept for other studies), and the captured DNA is sequenced. Direct shotgun sequencing of eDNA samples without prior amplification or target enrichment is, at least currently, an inefficient approach for the detection of eukaryotic diversity due to the large amount of nontarget DNA (Stat et al., 2017).

While multiple companies (i.e., Agilent, myBaits, and Roche) offer a selection of predesigned “off‐the‐shelf” probe kits, custom‐designed probes targeting specific regions of interest can be synthesized for purchase as well, albeit at a higher price. This provides applicability at a wide range of taxonomic levels, depending on the chosen design (e.g., ultra‐conserved elements (UCEs), exome capture, introns and intergenic regions, or previously identified RAD loci). Although a recent attempt to apply target capture to nuclear eDNA for population genetic analyses was unsuccessful (Pinfield et al., 2019), initial eDNA concentrations in this study were very low, and we therefore still believe that this approach holds promise for future eDNA research. For instance, an intriguing question for eDNA research is whether eDNA samples contain enough information about sequence differences in exonic regions to allow for the study of functional genetic variation, including changes in functional variation over time (Bálint et al., 2018). In human genetics, exome capture followed by high‐throughput sequencing is already widely used for detecting functional genomic variation, both in clinical diagnostics (Yang et al., 2013) and in basic research (Xu et al., 2011). To assess the potential applicability of similar inference based on eDNA samples, studies targeting exonic regions ought to be carried out under controlled conditions, sequencing the same group of individuals based on tissue samples and eDNA in parallel. Should it prove successful, such an approach would have wide applicability both in biological research and in commercial contexts, such as aquaculture, where genomic approaches are used to study functional variation in performance traits (Liu, 2003; Macqueen et al., 2017). While it remains unlikely that it will be possible to assign genotypes to specific individuals in this framework, different groups of individuals, such as different generations of individuals at an aquaculture facility, could be compared with respect to functional variation.

It is important to consider that, in contrast to tissue‐based analysis, the samples used in eDNA studies may also contain DNA from close relatives of the target species. In these cases, the probe design must rely on extensive reference sequence data, ideally genomes, of not only the target species, but also all locally occurring close relatives of the target species, to ensure the best possible species specificity of the technique. This presents a major challenge for eDNA‐based population genomics, and in the early stages of developing this field of study, the target capture approach may mostly be relevant in cases where the species of interest can be confidently assumed to be the only locally occurring species within its genus or family. Fundamentally, however, the challenge of taxonomic specificity is one that is faced in every eDNA study and has been successfully addressed before (e.g., Wilcox et al., 2013).

A consequence of the fragmented nature of eDNA is that it cannot provide multilocus genotypes (although see section 2.4), which means that the application of eDNA to population assignment of individuals and detection of individual admixture and hybridization is an unlikely prospect. However, if a large enough number of individuals contribute to eDNA samples, it should be possible to obtain reasonably accurate nuclear allele frequency estimates for the population as a whole (as demonstrated for mitochondrial DNA, Sigsgaard et al., 2016). This would allow for the application of mixed stock analysis (MSA) (Grant, Milner, Krasnowski, & Utterer, 1980), which is an important tool in fisheries management, and for demographic history analyses. Recently, population genetic studies requiring large sample sizes and/or based on organisms of small body size with low individual DNA yield have increasingly turned to pooled sequencing of tissue samples as an alternative method of reducing costs while obtaining reliable estimates of allele frequencies (Gautier et al., 2013; Schlötterer, Tobler, Kofler, & Nolte, 2014). In keeping with these developments, analytical approaches originally designed for individual sample data have been adapted to a pooled sequencing approach (Boitard et al., 2013). Importantly, population genetics based on eDNA more closely resembles such pooled sequencing of many individuals from a known population (as discussed by Bálint et al., 2018) than it does conventional sequencing of samples from individual organisms. Therefore, the theoretical and analytical framework developed for pooled tissue samples could potentially be of use to studies of eDNA samples. In tissue‐based studies, it has been recommended that a minimum of 50–100 individuals are pooled to ensure reliable estimation of allele frequencies (Lynch, Bost, Wilson, Maruki, & Harrison, 2014; Schlötterer et al., 2014), but several studies have found that smaller numbers of individuals may be sufficient (Gautier et al., 2013; Hivert, Leblois, Petit, Gautier, & Vitalis, 2018; Rode et al., 2018). However, as the variability of individual DNA contributions will likely be higher for eDNA samples than they are for tissue samples, where DNA concentrations of individual samples can be measured and adjusted before pooling, more individuals may be needed when working with eDNA. While sufficient numbers of individuals may not always be easily obtained from aquatic eDNA samples, it ought to be possible at least for species that display seasonal aggregations (Rowat & Brooks, 2012), mass spawning (Smith, 1972), or other schooling behavior (Gallego & Heath, 1994). Furthermore, combining several eDNA samples from the same area/population is also a possible solution to this problem, although it does come with a risk of diluting rare alleles (present in just a single or very few samples) to below detectability. Applying the theoretical framework, including statistical methods (e.g., models of allele frequency estimation accuracy, Rode et al., 2018), from pooled tissue samples to studies of eDNA samples, may thus yield significant advantages. However, caution is warranted until sufficient experimental validation has been carried out, both with mesocosm experiments and under natural conditions. Such validation experiments should at minimum include a comparison between pooled sequencing of tissue samples from individual animals in a mesocosm and sequencing of eDNA samples from the same mesocosm, as well as a comparison of eDNA samples from a natural environment with tissue samples collected immediately after eDNA sampling in the same area. Specifically, the main sources of variation influencing the precision of allele frequency estimation, that is (a) sampling variability—*the variability associated with the number of individuals sequenced, combined with the actual frequency of the studied alleles in the population*, (b) DNA pooling variability—*the variability that arises from unequal individual contributions to the DNA pool,* and (c) sequencing variability—*the variability associated with library preparation and sequencing itself* (Rode et al., 2018), must be investigated in the context of eDNA analysis.

### 5.2 Determination of sex ratios

While eDNA‐based population genetic analysis presents some important advantages over traditional tissue‐based approaches, a major advantage of the traditional methods is the possibility of pairing features of the study organisms, such as phenotypic traits, size, sex, age/developmental stage, and health condition, with the genetic data (Schmidt et al., 2009). However, some of this information may also be accessible through eDNA. For instance, sex ratios could potentially be estimated using sex chromosome markers. Based on the apparent correlations between seawater eDNA sequencing read abundance and the abundance of marine organisms (Sigsgaard et al., 2016; Thomsen, Møller, et al., 2016), the relative read abundance between sex‐specific markers alone might be informative. If applying a target capture and shotgun‐sequencing approach, relative read coverage for the different sex chromosomes might be used as a proxy for relative abundance of the sexes, as is done for the estimation of relative population abundance in microbiology (Albertsen et al., 2013). An evident shortcoming to this approach is that sexual systems are not always (at least not exclusively) chromosomally determined in vertebrates. Some reptiles carry no sex chromosomes, instead employing temperature‐dependent sex determination (Janzen & Paukstis, 1991; see Janzen & Phillips, 2006, for a mini‐review on environmental sex determination). It has been shown that even when sex chromosomes are present in these animals, temperature can sometimes overrule the genetic sex (Radder, Quinn, Georges, Sarre, & Shine, 2008). Certain fishes also employ environmentally dependent sex determination (Conover & Kynard, 1981; Ospina‐Álvarez & Piferrer, 2008) and intrinsic factors such as growth and behavior may affect sex differentiation in some species (see Devlin and Nagahama (2002) for a review on sex determination in fishes). The feasibility of detecting sex ratios from eDNA will therefore depend on the specific species in question. This said, epigenetic approaches (these will be discussed in section 5.4) could potentially still allow for sex ratio estimation from eDNA, namely in those cases where differential DNA methylation of specific genes is responsible for determining sex (Navarro‐Martín et al., 2011). One potential use of such a method could be to monitor the performance of artificial sex determination efforts in aquaculture, where monosex stocks are cultured, for example, to avoid undesired reproduction (see Cnaani & Levavi‐Sivan, 2009).

### 5.3 Identification of individuals

While traditional population genetic techniques can distinguish between and count individuals of a species through methods such as microsatellite‐based DNA profiling (Palsbøll et al., 1997), the identification and/or quantification of individuals represents a challenge for eDNA approaches. As eDNA samples can contain DNA from several individuals, only polymorphisms occurring on the same sequencing read can be assumed to originate from the same individual. It is possible that if the sample contains DNA fragments of sufficient length, then (small) linked groups of SNPs originating from the same individual could be identified by for instance linked read sequencing, where a unique barcode is added to every short read produced from the same individual DNA molecule (Zheng et al., 2016). If taxonomically specific cells of interest could be isolated efficiently by a cell sorting approach, single‐cell sequencing (Macaulay & Voet, 2014) could also potentially allow for unambiguous differentiation of individuals. Alternatively, differential read coverage could potentially help discriminate and assign sequences to different individuals, such as has been done for the identification of separate bacterial populations differing in relative abundance (Albertsen et al., 2013). The relative abundance of eDNA in a sample can be expected to differ between individuals, and the read coverage for sequences from an individual representing a small part of the eDNA pool will thus be expected to be consistently lower than the read coverage for sequences from an individual with a high contribution of DNA to the environment. In combination with epigenetic techniques, these approaches might even make it possible to determine characteristics such as age and indications of health (Horvath & Raj, 2018; Park, Friso, & Choi, 2012; Shimoda et al., 2014) of different individuals represented in an eDNA sample. Such methodologies must of course be experimentally validated, but in the meanwhile the total amount of allelic diversity in the eDNA data could in itself provide an indirect estimate of the local abundance of a species (as mentioned above in the section on mtDNA), which is valuable information for the management and conservation purposes. Importantly, many useful inferences can be made without the need for distinguishing between individuals, and for species with very large populations (this includes most common marine fishes) individual identification is nowhere near as relevant as it is for, for example, whales or large sharks.

In the case of such abundant species, the employment of an intense sequencing effort and a highly variable marker (e.g., one containing linked microsatellites) might make it possible to use a rarefaction method to estimate the total number of genotypes in an area and thereby estimate the census population size (Eggert, Eggert, & Woodruff, 2003). For instance, capture probes targeting the flanking regions of short microsatellite regions (Kistler et al., 2017) or of transposable elements (Rey‐Iglesia et al., 2019) could be designed, and the allelic richness obtained in the captured sequences could then be used as a direct estimate of the minimum number of contributing individuals, and indirectly in a rarefaction approach, to estimate total population size (Eggert et al., 2003). This method would be especially suited for highly polymorphic species with plenty of genetic reference information available, such as *Gadus morhua* Linnaeus, 1758, (Atlantic cod) (Star et al., 2011) or the panmictic eels *Anguilla anguilla* (Linnaeus, 1758) (European eel) and *A. rostrata* (Le Sueur, 1817) (American eel) (Als et al., 2011; Côté et al., 2013; Pavey et al., 2017), where parameters of great economic interest, such as the minimum number of individuals in a cod aggregation or of eel larvae in an area of the Sargasso Sea, could be assessed. It should be noted, however, that designing probes immediately adjacent to microsatellite regions may compromise probe quality, as these are often affected by problems such as high‐sequence complexity and low GC content (Cruz‐Dávalos et al., 2017; Ellegren, 2004), which cause an increase in amplification and sequencing error rates. Alternatively, simply targeting intronic or intergenic regions for capture might provide equally accurate estimates without the need for extensive reference data. In light of the high PCR and sequencing error rates associated with microsatellites, this latter approach may prove more attractive for most eDNA studies. An additional note of some importance is that if multiple microsatellites are located close enough in the genome to be contained in a single read (the length of which, on currently dominant sequencing technologies, does not exceed 1,000 bp), they would most likely be affected by strong linkage disequilibrium (nonrandom association of alleles at different loci, Slatkin, 2008) and may therefore not be sufficiently independent for use in robust population genetic analyses. However, the budding era of long‐read sequencing, with currently advertised lengths of up to 900 kb (Oxford Nanopore Technologies), may ameliorate this problem in the not‐so‐distant future.

### 5.4 Epigenetics

Environmental DNA methods may eventually enter the rapidly expanding field of epigenetics—the study of changes in eukaryotic organisms caused by the modification of gene expression rather than mutations in the genes themselves. In particular, differential methylation patterns in the nuclear genome have received much attention in a wide range of biological fields over the recent years. DNA methylation occurs primarily through the enzyme‐catalyzed transfer of a methyl group to cytosine residues, which can be detected by employing bisulfite sequencing (Gatzmann & Lyko, 2019), antibodies (Morimoto et al., 2017), SMRT sequencing (Flusberg et al., 2010), or nanopore technology (Simpson et al., 2017). While still an incipient field, we are beginning to understand how factors such as aging or environmental stress are reflected in methylation patterns of the genome in humans (Horvath & Raj, 2018) and other animals, such as fish (Aluru, Karchner, Krick, Zhu, & Liu, 2018; Moghadam et al., 2017; Shimoda et al., 2014) and insects (Srinivasan & Brisson, 2012). Even for nonmodel species, development of epigenetic markers has been used to determine the age of individuals with remarkable precision (Paoli‐Iseppi et al., 2019). Based on these results, PCR primers targeting specific methylation sites with known associations to physiological state could potentially be designed for application on eDNA samples. However, genomic methylation patterns can differ between tissue types (Zhang et al., 2013), and the tissue type from which the sampled eDNA sequences originate from would be initially unknown. Thus, potential target sites would be limited to sites known to show the same methylation response across tissue types, or to be uniquely methylated in certain tissue types only. Prior information from tissue‐based studies would need to be available, not only for the specific species, but it would have to expand into tissue‐specific reference information, covering at least the most likely source tissue types for eDNA (for vertebrates, this would include epithelial cells from the skin, gut, and urinary system), as well as different age groups. While freshly shed eDNA will still be methylated, methylated cytosines are gradually deaminated during DNA degradation. This results in transitions from methylated cytosine to thymine residues, but when sufficient genomic reference sequences are available for alignment, these transitions can be recognized as the result of methylation, and methylation patterns can thus still be indirectly obtained, even for ancient DNA thousands of years old (Llamas et al., 2012). With regard to the prospect of using eDNA for epigenetic studies, direct detection of methylation using, for example, bisulfite sequencing might have to be combined with the detection of deaminated cytosines to ensure that an observed deficit of methylation is not a by‐product of deamination. In the latter process, bioinformatic tools, already developed for ancient DNA studies, could be applied to distinguish between suspected transitions that are true variants, or the result of sequencing errors, or indeed of deamination. If (or when) such epigenetic techniques are adapted for application on eDNA samples, they would open up some exciting avenues of research, such as the possibility of remotely assessing the level of environmental stress a given population is currently experiencing. Furthermore, if potentially differential levels of eDNA shedding between juveniles and adults can be taken into account, perhaps one will also have the possibility to infer relative age composition in a noninvasive manner.

### 5.5 Environmental RNA and gene expression

Due to the instability of RNA molecules in vitro, RNA has generally been expected to rapidly degrade in the environment, and environmental RNA (eRNA) has therefore received less attention than eDNA as markers for biodiversity monitoring (Cristescu, 2019). On the other hand, the faster degradation rate has also been suggested to offer the advantage of a more accurate spatiotemporal picture of biotic communities than eDNA, as eRNA is expected to reflect only currently living cells, and long‐distance transport of the molecules is thought to be limited (Cristescu, 2019; Laroche et al., 2016; Lejzerowicz et al., 2015; Pochon, Zaiko, Fletcher, Laroche, & Wood, 2017). In support of this, there is some evidence that eRNA is more strongly correlated with environmental variables (Laroche et al., 2016) and morphological diversity indices (Pochon et al., 2017) than eDNA (although see Keeley, Wood, & Pochon, 2018). This could bring up concerns that eRNA concentrations are too low for population‐level studies, but some studies have shown that RNA can, under the right circumstances, persist for long periods of time (Fordyce et al., 2013), perhaps through protection within extracellular vesicles (Kim, Abdelmohsen, Mustapic, Kapogiannis, & Gorospe, 2017; Koga et al., 2011) or protein capsids (Ashley et al., 2018), and other recent studies indicate that organisms can sometimes release very large amounts of RNA into the environment (reviewed by Cristescu, 2019). Thus, eRNA may in some cases be present in sufficient amounts in the water column to allow for remote studies of real‐time differential gene expression (including both differences in overall transcription rates and splicing variation) between populations of aquatic macroorganisms.

## 6 CHALLENGES AND REMAINING QUESTIONS

**Figure 1:**
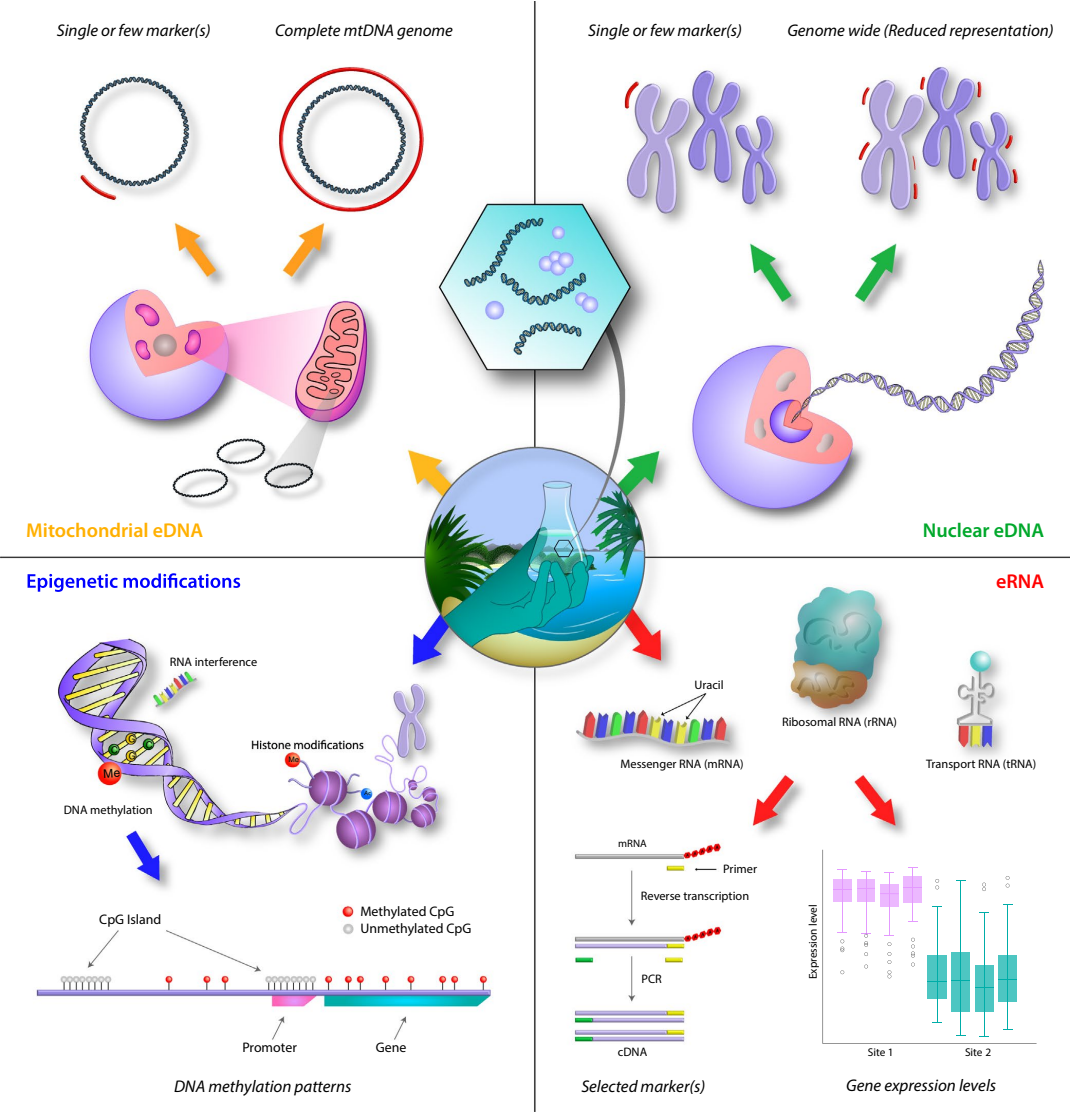
An overview of current and potential future uses of eDNA from water samples for studying population genetics of macroorganisms

While eDNA potentially offers a wide range of valuable applications in population genetic research (Figure 1), these approaches also come with associated challenges. Some of these challenges apply to both mtDNA and nuDNA, and to genome‐wide as well as single‐marker approaches. These include, but are not limited to, (a) PCR and/or sequencing errors leading to false‐positive detections of haplotypes (Oliver, Brown, Callaham, & Jumpponen, 2015); (b) allelic dropout due to low‐abundant or fragmented DNA (Smith & Wang, 2014); (c) relative read abundances may not reflect individual abundances, due to, for example, different eDNA shedding rates between individuals, and biased PCR amplification or capture efficiency (e.g., Alberdi, Aizpurua, Gilbert, & Bohmann, 2018; Elbrecht & Leese, 2015); (d) co‐amplification/co‐capture of DNA from closely related species (Burbano et al., 2010; Wilcox et al., 2013); (e) risk of removing true genetic variation during bioinformatic filtering (Alberdi et al., 2018); (f) unknown number of individuals contributing to the eDNA pool (Sigsgaard et al., 2016) and difficulty assigning sequences from multiple markers to individuals (Adams et al., 2019); and (g) heteroplasmy (different organelle genomes within the same cell or the same individual) leading to overestimation of genetic diversity and number of individuals (Shokralla et al., 2014).

The errors introduced in the raw sequence data during PCR and sequencing are currently a major challenge for eDNA analyses, as this can lead to false‐positive detections of haplotypes (Taberlet et al., 2018). Even when a good reference panel of haplotypes is available, it may be incomplete, and it is therefore critical to be able to distinguish between sequences that are likely erroneous and sequences that are previously unknown, but likely to be true haplotypes. This challenge is especially relevant for long‐read sequencing technologies, such as nanopore and SMRT sequencing, where error rates are still relatively high (Laver et al., 2015; Weirather et al., 2017), although so‐called hybrid sequencing, which combines data from long‐ and short‐read sequencing platforms, has proven a good strategy for simultaneously obtaining long and high‐quality sequences (Goodwin et al., 2015; Laver et al., 2015).

One way to identify potential errors is to include in the experiment a positive control consisting of tissue samples of known haplotypes and use it to estimate the sequencing error rates. This allows the bioinformatic pipeline to filter out any eDNA sequences that have an abundance at or below the random error rates observed in the control sample (Adams et al., 2019; Parsons et al., 2018; Sigsgaard et al., 2016). A more sophisticated filtering approach can be used with bioinformatic software that groups similar eDNA sequences into clusters and removes sequences below a certain threshold of abundance relative to potential “source sequences.” This can be done using a model with pre‐set parameter values (Boyer et al., 2016; Edgar & Flyvbjerg, 2015), or using error models based on the eDNA sequencing data itself (e.g., Callahan et al., 2016). If a coding region is used for metabarcoding, likely errors and suitable filtering thresholds can also be identified based on changes in entropy of the different codon positions, as shown for community DNA samples (Turon, Antich, Palacín, Præbel, & Wangensteen, 2019). Errors can also be reduced by limiting PCR amplification, for instance by using target capture.

Using analytical frameworks that incorporate genotype likelihoods (Korneliussen, Albrechtsen, & Nielsen, 2014; reviewed by Nielsen, Paul, Albrechtsen, & Song, 2011), an approach that is currently increasing in popularity in population genetics, instead of traditionally called genotypes, would also help to alleviate the problem of false haplotypes introduced by sequencing errors. Similarly, given an appropriate reference database, phylogeny‐based software can taxonomically classify DNA sequences while providing statistically meaningful measures of confidence (Munch, Boomsma, Huelsenbeck, Willerslev, & Nielsen, 2008; Somervuo, Koskela, Pennanen, Henrik Nilsson, & Ovaskainen, 2016). For instance, the software PROTAX takes into account taxa that are present in the taxonomy, but do not have reference sequences, as well as the possibility of unknown taxonomic units and mislabeled reference sequences (Somervuo et al., 2016). Thus, using a database of known haplotype variants for a target species, such software could be applied to obtain probabilities of eDNA sequences being true haplotypes.

Importantly, very strict filtering may lead to dismissal of true genetic variation (Taberlet et al., 2018). For instance, some true sequences may consistently yield low‐quality sequence reads, due to, for example, repeats in the sequence as suggested by Taberlet et al. (2018). Rare alleles may also be lost due to very low concentrations in the eDNA pool or a high degree of fragmentation (Smith & Wang, 2014). Encouragingly, depending on the research question, a small number of false‐negative or false‐positive haplotype detections may have little or no influence on the reliability of the final conclusions. For instance, one measure of genetic diversity widely used to estimate long‐term effective population size is the overall average number of nucleotide differences between two DNA sequences in the population (Tajima, 1983), making it quite robust against the influence of a few rare sequences (e.g., Sigsgaard et al., 2016). This consideration will be essential for future research, as reference databases for eDNA data are still far from complete, and will (to some degree) remain so for the foreseeable future, only containing sufficient levels of information on intraspecific variation for a short list of species.

A factor likely to present a greater challenge than amplification and sequencing errors are the potential biases affecting the correlation between relative abundance of alleles/haplotypes in the population and respective eDNA read abundances, which could in turn bias certain population genetic analyses dependent on reliable estimates of low‐frequency alleles/haplotypes, such as analysis of allele frequency spectra (Gutenkunst, Hernandez, Williamson, & Bustamante, 2009). For instance, it should be considered that juvenile animals may shed eDNA at higher rates per biomass relative to adult individuals, due to increased cell turnover during growth and development. Adult individuals, on the other hand, may shed a larger total amount of eDNA due to their larger body size (Maruyama, Nakamura, Yamanaka, Kondoh, & Minamoto, 2014). If allele frequency differences exist between cohorts, for example, due to strong drift or different populations having contributed to different cohorts within a site (Knutsen et al., 2018), then problems of reliably estimating allele frequencies might be exacerbated at the eDNA level. Similarly, feeding activity and diet can affect eDNA shedding rates (Klymus, Richter, Chapman, & Paukert, 2015) and may differ between individuals and cohorts. Thus, the age distribution of the population, as well as the diet, activity level, and biomass of individuals, could potentially impact the accuracy of eDNA‐based population genetic analyses. Lastly, the transport, dispersion, and degradation of eDNA may differ between habitats (Thomsen & Willerslev, 2015), which may result in a shorter or longer “catch‐window” for detecting certain groups of individuals. To investigate the extent of these possible biases, as well as to address questions regarding the density of sampling needed under different circumstances, further mesocosm experiments and comparisons with traditional approaches are a pressing priority for the development of this field.

Specifically, for genomic approaches based on nuclear eDNA, major challenges include (a) relatively small amounts of template DNA in comparison with mtDNA, (b) a large gap in the reference databases for genomes as compared to mtDNA; and (c) expensive techniques (capture probes). In the meantime, until these challenges are dealt with, nuDNA might still in some cases offer advantages in single‐marker/metabarcoding approaches; for instance, the ribosomal RNA gene internal transcribed spacer‐1 (ITS‐1) has been found to vary at the intraspecific level (Wang et al., 2015) and could thus prove useful for eDNA‐based population genetic studies. This gene exists in multiple copies in the nuclear genome and has been found to be a more sensitive marker for *Cyprinus carpio* (common carp) than the mitochondrial Cyt*b* gene (Minamoto et al., 2017). In contrast to mitochondrial markers, nuclear genes are also expected to exist in the same number of copies across cell types (Long & Dawid, 1980) and this predictability might offer more accurate estimates of biomass and abundance of aquatic organisms.

While species such as the whale shark offer optimal conditions for eDNA sampling due to their seasonal aggregation behavior, species which are seldom or never found in larger groups may require very intensive sampling to obtain sufficient coverage of the genetic diversity in the population. This problem may be ameliorated by collecting samples as specifically as possible in places where the animals have been observed, such as by sampling fluke prints from porpoises (Parsons et al., 2018), or places where local conditions are known to be favorable for the species. This would also minimize the risk of sampling eDNA from closely related species, which may complicate subsequent analyses if these species are co‐amplified or co‐captured together with the target species. In cases where the number of individuals in the sampling area is small and the individuals can be easily observed, the number of source individuals for each eDNA sample can be closely estimated, offering an advantage compared with the many cases where the number of individuals contributing to a sample will be unknown. However, the use of eDNA would in such cases offer limited advantages compared with tissue sampling, and the latter might be preferred, especially if additional experiments such as isotope analyses are of interest for the same samples.

While the co‐occurrence of DNA from several species in eDNA samples may cause problems for population genetic analyses, this same characteristic of eDNA may also offer insights, which are not possible to deduce from tissue samples. For instance, a single sample set may be used to study not just the individuals of the population of interest, but also co‐occurring biodiversity such as prey species, symbionts, or diseases (Sengupta et al., 2019). A single metabarcoding assay may even be applied to study both inter‐ and intraspecific diversity of a group of organisms, such as fish (Stat et al., 2017), or even across whole communities of eukaryotes simultaneously (Turon et al., 2019). Lastly, the relative ease and cost efficiency of sampling offers a range of opportunities for long‐term temporal studies of communities and populations (Devictor et al., 2012; Thomsen, Jørgensen, et al., 2016; Warren et al., 2001), a type of study, which is currently rare (Magurran et al., 2010). Environmental DNA studies could thus offer a valuable source of information on temporal dynamics not just of aquatic communities (Sigsgaard et al., 2017; Stoeckle, Soboleva, & Charlop‐Powers, 2017; Ushio et al., 2017), but also of populations (Bálint et al., 2018), including for instance year‐to‐year or even season‐to‐season fluctuations in population size and sex ratios.

## 7 CONCLUDING REMARKS

Environmental DNA from seawater samples has shown a lot of potential as a noninvasive approach to study the population genetics of marine vertebrates, using short mitochondrial markers. However, as we have outlined here, if modern techniques developed in related fields, such as human genomics, are applied to aquatic eDNA samples, the approach could eventually be expected to provide not just an increased resolution in population genetic inference, but also additional types of data, such as genome‐wide SNP data, and physiologically important information on epigenetic patterns and gene expression. Lastly, an eDNA approach can offer ecological insights that are not accessible with traditional tissue samples, by simultaneously providing population genetic information on the target organism and the presence/absence or abundance information on co‐occurring organisms (Sigsgaard et al., 2016; Stat et al., 2017). Importantly, the techniques outlined here could potentially be applied to all aquatic macroorganisms, as well as to many other complex sample types, including bulk samples (Yu et al., 2012), soil (Zinger et al., 2018), and plant material (Monge, Dumas, & Baus, 2018; Thomsen & Sigsgaard, 2019), blood meals from invertebrates (Schnell et al., 2012), and fecal samples (Hibert et al., 2013). All these applications stand to benefit greatly from the current expansion of reference databases, such as the National Center for Biotechnology Information's (NCBI) Genbank and the Barcode of Life Database (BOLD), to include complete genomes for a greater number of species and to more exhaustively cover inter‐ and intraspecific variation, developments that have been accelerating in recent years. As discussed, while certain vertebrates lend themselves well to the eDNA approach by, for instance, forming large feeding aggregations, other species may require a large and well‐planned sampling effort to obtain eDNA from a sufficient number of individuals. Thus, determining the minimum level of sampling necessary for generating reproducible results, as well as outlining under which circumstances eDNA analysis constitutes an advantageous approach compared with alternative approaches, is of high priority for future research. However, at least for endangered, elusive, and economically important species, eDNA‐based population genetic methods offer an attractive avenue for improved monitoring and biological research.

## CONFLICT OF INTEREST

The authors declare no conflicts of interest.

## GLOSSARY

Bisulfite sequencing: Treatment of DNA with bisulfite before sequencing to determine methylation patterns. Bisulfite treatment converts unmethylated cytosine residues to uracil, but does not affect methylated cytosine residues.
Capture probe: Short synthesized oligonucleotides typically 55‐120 bp in length (Clark et al. 2011; Sulonen et al. 2011) designed to hybridize to specific DNA sequences. They are bound to a surface, thereby facilitating the targeted capture of certain sequences in a sample.
DNA methylation: The addition of methyl groups to DNA. Occurs primarily through the enzyme‐catalyzed transfer of a methyl group to cytosine residues.
Exome capture: Capture approach targeting all exons across the genome.
High‐throughput sequencing (HTS): Simultaneous sequencing of thousands to billions of DNA fragments or amplicons.
DNA metabarcoding: The simultaneous identification of several taxa in a complex sample, by amplifying and sequencing a short genetic region known as a DNA barcode
Reduced representation library (RRL): A sequencing library consisting of short genetic regions from across the nuclear genome, yielding a large number of (more or less) independent sites for comparisons across individuals and populations.
Restriction site‐associated DNA sequencing (RADseq): A sequencing approach where restriction enzymes are used to cut DNA into fragments, which are then tagged with molecular identifiers unique to each individual and sequenced in high throughput.
Sequencing by synthesis (SBS): A sequencing approach based on the use of modified nucleotides, which are marked with a fluorescent dye specific to each of the four bases, and contain a reversible blocker that blocks further incorporation of nucleotides until removed chemically.
Shotgun sequencing: The sequencing of DNA, which has been randomly sheared into fragments.
Single‐molecule real‐time sequencing (SMRT): A parallelized sequencing approach, where single DNA molecules are isolated in small structures called zero‐mode waveguides together with a single polymerase enzyme. A detector can then observe the incorporation of each single fluorescently labeled single nucleotide.
Single nucleotide polymorphism (SNP): Site in the genome that varies between individuals in a population by a single nucleotide substitution.
Target capture: Targeted enrichment of DNA based on hybridization to capture probes.
Ultra‐conserved element (UCE): Highly conserved regions in the genome, flanked by more variable sequences.
Whole‐genome sequencing (WGS): Sequencing of an organism's complete genome in a single experiment.

## Data Availability

Data sharing is not applicable to this article as no new data were created or analyzed in this study.
